# Bio-based polyurethane foam preparation employing lignin from corn stalk enzymatic hydrolysis residues†

**DOI:** 10.1039/c8ra01089g

**Published:** 2018-04-26

**Authors:** Shiyun Zhu, Kefu Chen, Jun Xu, Jun Li, Lihuan Mo

**Affiliations:** State Key Laboratory of Pulp and Paper Engineering, South China University of Technology Guangzhou Guangdong 510640 China xujun@scut.edu.cn +8613828470869; Plant Micro/Nano Fiber Research Center, South China University of Technology Guangzhou 510640 Guangdong China

## Abstract

Enzymatic hydrolysis residues (EHR) from corn stalk are industrial waste from the cellulosic ethanol industry. Lignin was separated as a bio-based polyol from EHR replacing partial petroleum-based polyether polyol to prepare bio-based polyurethane (BPU) foams without any other biomass pretreatment. Single factor experiment and response surface methodology (RSM) were employed to optimize separation conditions and reveal the significant influence of the interaction of conditions on the yield of separated lignin (SL). The effect of SL content (2.5, 5.0, 7.5, 10 and 15%) on the foams morphology and mechanical properties was assessed. Scanning electron microscopy (SEM) results implied that the cell shape was considerably affected by the large SL content, which contributed to an irregular, inhomogeneous, and thick cell wall. An astonishing 9.56 times increase in the compressive modulus and exponential 97.93 times boost in the compressive strength of BPU foams were attributed to the content of 15% SL without any further surface chemical modification. This present paper reports a green, potential and promising method for complete utilization of lignin from EHR in consideration of their abundant supply to greatly enhance the mechanical properties of BPU foams.

## Abstract 2

Enzymatic hydrolysis residues (EHR) from corn stalk are industrial waste from the cellulosic ethanol industry.

## Graphical abstract

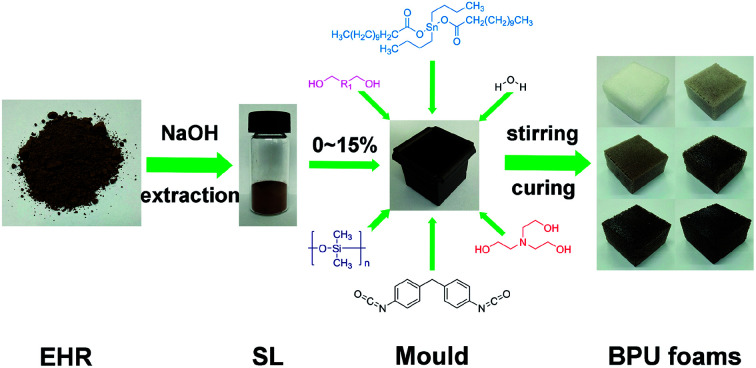

## 1. Introduction

Polyurethanes (PU) are a versatile class of polymeric materials exhibiting a broad range of applications such as furniture, automotive seating, thermal insulators, packaging and medical devices in form of foams, adhesives, elastomers and many others. PU foams are synthesized by a polyaddition reaction between polyols and isocyanates in addition to other additives such as surfactants and catalysts used to balance the reaction in the forming process.^1^ However, most of these reagents of PU foams are derived from petroleum, increasing the fossil-fuel source dependence and environmental problems due to its status as a limited resource.^2^ Therefore, biodegradable and renewable bio-based raw materials are receiving increasing attention and popularity.

Recently, the bio-based polyols have been investigated for the production of sustainable and eco-friendly PU foams, such as lignin,^3^ castor oil,^4,5^ cellulose,^6^*etc.* Specially, lignin is an important and desirable candidate to replace petroleum-based polyols partially or totally for the production of bio-based PU foams due to its renewability, low cost, abundance and unique chemical structure.^7,8^ As a promising source for sustainable and bio-base polymeric materials, lignin offers perspectives for higher-added-value applications such as flexible, semi-rigid, rigid PU foams.^9,10^

Lignin is arguably the second most abundant biopolymer on earth with cross-linked aromatic structure contained many phenolic and aliphatic hydroxyl groups.^11–13^ Currently, most of the lignin considered as by-product is burned to recover heat and electricity. Only 1–2% of the annually produced lignin is being commercialized for the preparation of bio-chemicals and to limited extent for bio-materials.^14,15^ Lignin is mainly obtained from black liquor or as a residue of cellulosic ethanol production process.^7,16^

In previous studies, lignosulfonate and hydrolyzed lignosulfonate under alkaline conditions were used as polyol components in polyurethane foam preparation.^17^ Haemin Gang successfully produced bio-based polyurethane foam (BPU) elastomers using vanillin-based polyol chain extender.^18^ However, the bio-based polyols are not easy to apply into industrial-scale production due to the high cost or complex process.

Several attempts have made to isolate lignin from residues or black liquor. Guo *et al.* reported a system in separating lignin from bio-ethanol production residue with organic solvents (benzyl alcohol, dioxane, ethanol).^19^ Bouxin *et al.* have extracted lignin by a two-step process combining percolation-mode ammonia pretreatment with mild organosolv purification.^20^ Lee suggested that lignin was separated from the residue of bioethanol production with oak wood *via* alkaline and catalyzed organosolv treatments at ambient temperature.^21^ Liu *et al.* reported that lithium chloride (LiCl) and dimethyl sulfoxide (DMSO) solvent was applied prior to enzymatic hydrolysis to isolate lignin for higher yield and smaller structural changes.^22^ Nevertheless, the above separation methods are not easy to operate and dangerous or toxicant. A relatively simple and secure method is significant for better separation of lignin from enzymatic hydrolysis residues. Thus, a more moderate alkali–acid treatment without any organic solvent extraction nor high temperature and pressure was applied using the published procedures^23^ with some modifications.

Recent studies indicated that lignin could be liquefied, modified chemically (lignin oxypropylation) and combined with other materials (castor oil, graphene oxide) for the preparation of BPU foams.^5,24–27^ Furthermore, most of these hybrid polyols production methods require more energy (mainly heat or microwave) and reagent consumption to homogenize the system phases.^7,28^ Consequently, a simple, green and low-cost approach was created to prepare BPU foams without loss of mechanical properties in this work. Lignin was directly mixed with polyether polyol without any liquefication, modification or incorporation. The hybrid polyols were just stirred uniformly without heating, ultrasonication or microwave treatment. In this study, lignin was separated from enzymatic hydrolysis residues *via* alkali-acid treatments and the separation process was optimized using response surface methodology. Separated lignin (SL) replacing partial polyether polyol was mixed with isocyanates, catalysts, blowing agent and surfactant to prepare BPU foams. Physical and mechanical properties such as density, compressive strength and modulus as well as thermal stability were investigated. Particularly, compressive strength and modulus of BPU foams were extremely excellent. Therefore, lignin used as a bio-polyol for the preparation of BPU foams could represent a superior utilization of a bio-refinery waste.

## 2. Materials and methods

### 2.1 Materials

EHR of corn stalk obtained from COFCO Bio-energy and Bio-chemical Co., Ltd. (Zhaodong, China) was dried, crushed and sifted to 40–60 mesh powder for lignin separation. Polyether 330n and 4,4′-diphenylmethane diisocyanate (MDI) were purchased from Wanhua chemical co., Ltd. (Yantai, China). Cholesterol, pyridine, deuterated chloroform (CDCl_3_) and chromium acetylacetonate were purchased from Macklin Biochemical Co., Ltd (Shanghai, China). 2-Chloro-4,4,5,5-tetramethyl-1,3,2-dioxaphospholane (TMDP) was purchased from Sigma-Aldrich. Polydimethyl siloxane (PDMS), triethanolamine (TEOA) and dibutyltin dilaurate (DBTDL) were used as received without further purification. All other reagents were of analytical grade. Deionized water was used throughout this study.

### 2.2 Separation of lignin

#### 2.2.1 Single factor experimental design

EHR powder was added into NaOH solution and the mixture was stirred in the water bath. Afterwards, the mixture was filtrated and diluted HCl was added into the filtrate to obtain the precipitation (pH = 1.5). After standing for 12 hours at room temperature, the precipitation was washed with deionized water, then filtrated and freeze-dried. Factors of concentration of NaOH, liquid–solid (L–S) ratio, temperature and time were investigated to determine the yield of SL in Table S1.† SL was acid insoluble and the yield, *Y* (%), was calculated according to the following equation:1
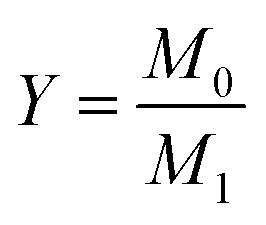
where *M*_0_ and *M*_l_ are the mass of acid insoluble lignin and EHR powder (g), respectively.

#### 2.2.2 Optimization *via* RSM

Preliminary experiments had been operated to sift suitable levels of each factor by single factor experimental design. Four factors *i.e.*, NaOH concentration (20–40, g L^−1^), L–S ratio (20 : 1–60 : 1, mL g^−1^), temperature (30–70, °C) and time (1.5–3.5, h) were investigated in single factor analysis. Then, four independent variables for the separation of lignin were optimized based on the selection of most significant parameters. Afterwards, RSM with Box–Behnken design was employed to analyze the interaction effects of four independent variables on the yield of lignin through the statistically designed experiments. All the levels of four factors were showed in Table S2,† where the levels of NaOH concentration, L–S ratio, temperature and time, coded as *A*, *B*, *C* and *D*, were in the range of 30–50 g L^−1^, 30 : 1–50 : 1 mL g^−1^, 50–70 °C and 2–3 h, respectively.

### 2.3 Preparation of BPU foams

**Fig. 1:**
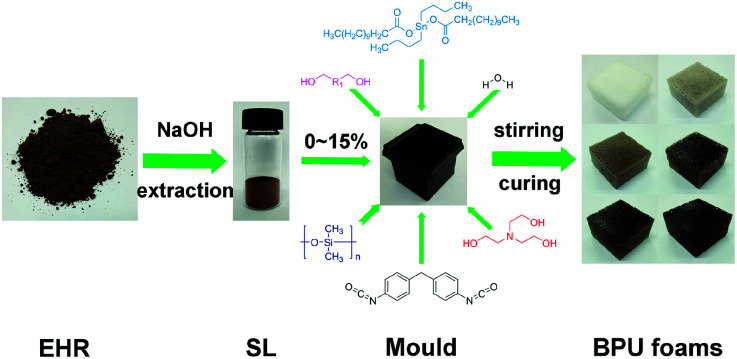
Procedures of the synthesis of BPU foams.

SL was previously grinded to 200 mesh particles and added into polyether polyol 330, then the mixture was stirred vigorously at an ambient temperature for 12 hours (1500 rpm). After the homogeneous dispersion of SL in polyether polyol, other additives such as catalyst (TEOA and DBTDL), blowing agent (water) and surfactant (PDMS) were added into the mixture, respectively. Afterwards, all ingredients were homogenized thoroughly in a 30 mL glass bottle using the electromagnetic stirring for 5 min (1500 rpm). Predetermined MDI was then added into the glass bottle and stirred rapidly until the bubble grew. The formulations were poured into a metal mould with the dimensions 50 × 50 × 50 mm for the growth of BPU foams. Afterwards, all BPU foams were cured for 12 h at 105 °C. The foam stability, cells morphology, shrinkage and structural uniformity could be observed at curing point. A summary process was shown in Fig. 1. Prior to further characterization, BPU foam samples were dried for 48 h at least depending on the testing requirements. BPU foams were synthesized using the batch process method as illustrated in Fig. S1† with different content of SL in Table 1.

**Table 1:** Formulation of BPU foams preparation

Ingredients	Weight (g)					
	Ref^a^	PF_2.5_^b^	PF_5_^c^	PF_7.5_^d^	PF_10_^e^	Opt^f^
SL	0	0.25	0.5	0.75	1.0	1.5
Polyether	10.0	9.75	9.5	9.25	9.0	8.5
Water	0.15	0.15	0.15	0.15	0.15	0.15
TEOA	0.10	0.10	0.10	0.10	0.10	0.10
DBTDL	0.12	0.12	0.12	0.12	0.12	0.12
PDMS	0.20	0.20	0.20	0.20	0.20	0.20
MDI	3.0	3.0	3.0	3.0	3.0	3.0
IN	1.73	1.25	0.98	0.81	0.69	0.53

a Ref: reference foam with 0 SL.

b PF_2.5_: BPU foam with 2.5% SL.

c PF_5_: BPU foam with 5% SL.

d PF_7.5_: BPU foam with 7.5% SL.

e PF_10_: BPU foam with 10% SL.

f Opt: Optimal foam with 15% SL.

### 2.4 Characterization of SL and BPU foams

The quantitative ^31^P-NMR analysis could detailedly describe the content of hydroxyl group in lignin. Dried SL powder (40 mg) was mixed with 2-chloro-4,4,5,5-tetramethyl-1,3,2-dioxaphospholane (TMDP, 130 μL) in a solution of pyridine/CDCl_3_ (1.6 : 1, v/v), chromium acetylace-tonate (relaxation agent), and cholesterol (internal standard). TMDP could react with aliphatic and phenolic hydroxyl groups as well as carboxylic acids groups in lignin in the presence of pyridine, which acted as the base in the solvent mixture to capture the liberated hydrogen chloride and drive the slightly exothermic overall phosphitylation reaction to total conversion.^29–31^ The NMR experiment was carried out on a Bruker DRX-400 NMR spectrometer at 400 MHz at 25 °C.

NCO/OH (isocyanate index, IN) required for the reaction is calculated using the following equation:2
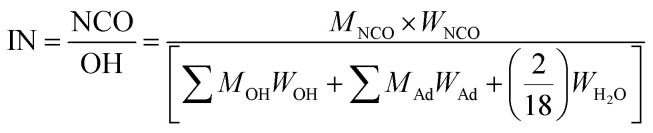
where *M*_NCO_ is the number of isocyanate groups in one gram of isocyanate, *W*_NCO_ is the weight of isocyanate (g), *M*_OH_ is the number of hydroxyl groups contained in one gram of polyols (mixture of separated lignin and polyether polyol), *W*_OH_ is the weight of polyols (g), *M*_Ad_ is the number of hydroxyl groups in one gram of additives (catalyst, and surfactant), *W*_Ad_ is the weight of additives (g) and *W*_H_2_O_ is the weight of water.

Fourier transform infrared (FT-IR) spectra were recorded on a VERTEX 70 Bruker spectrometer using an ATR accessory with resolution of 4 cm^−1^ in the range of 3700–560 cm^−1^. The foams were previously dried at 105 °C for 4 h to ensure that no water was adsorbed inside.

The thermal behavior of the foams was evaluated by thermogravimetric analysis (TGA), which was carried out on a TA equipment model STA449 F3 at temperatures ranging from 30 °C to 600 °C with a heating rate of 10 °C min^−1^ under a nitrogen flow.

The apparent density of BPU foams was calculated following the ASTM D 1622 standard by measuring three specimens of each sample with the dimensions of 50 × 50 × 25 mm. Results of ratio between weight (*M*) and volume (*V*) of the specimens was the apparent density, (kg m^−3^). Five specimens were tested for each foam sample, and the average value was calculated along with the standard deviation.

Mechanical testing of BPU foams was performed on a Tensile Compressive Universal Testing Machine INSTRON 5565 (USA) with the specimen size of 50 × 50 × 25 mm. At least five samples were analyzed to obtain the average value according to the ASTM D1621 standard. The measurement of compressive strength and modulus were conducted by compressing the specimen 10% of the thickness at 2.5 mm min^−1^ and determined the final stress value after 60 s of compression. Five specimens were tested for each foam sample, and the average value was calculated along with the standard deviation.

Samples were coated by a sputter-coating with evaporated gold, and subsequently, scanning electronic microscopy (SEM) was performed with a EVO 18 scanning microscope to observe and evaluate the cellular structure of the BPU foams.

## 3 Results and discussion

### 3.1 Single factor experiment

**Fig. 2:**
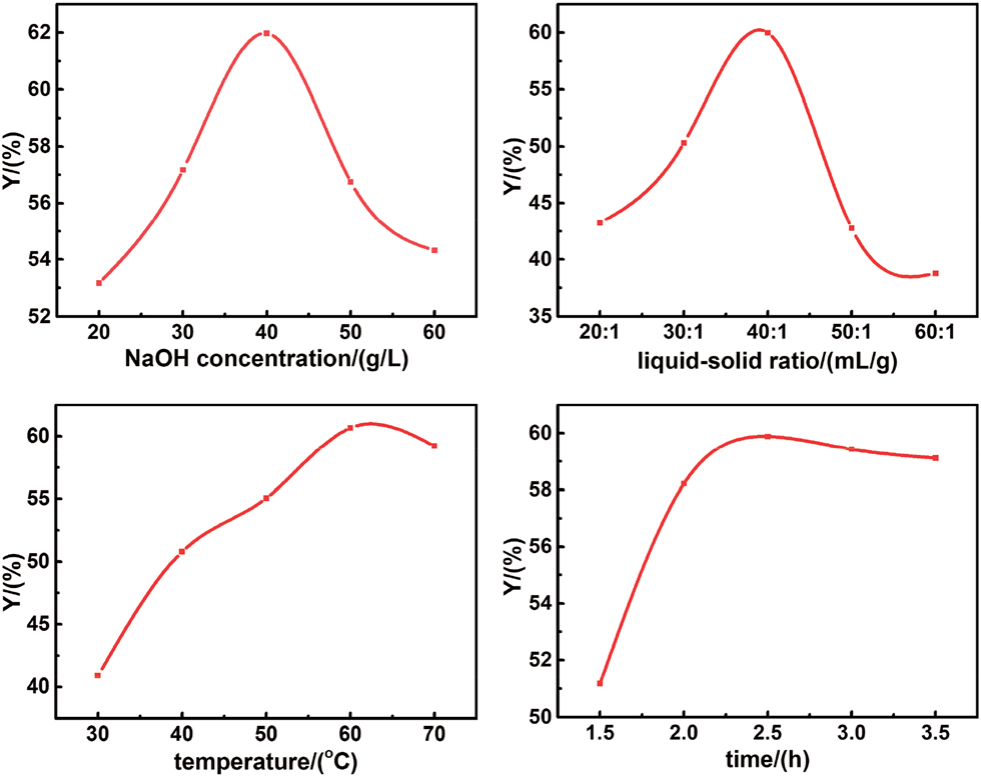
Effects of independent factor on the yield of SL.

Batch experiments were carried out in order to assess the effects of NaOH concentration, liquid–solid ratio, temperature and time on the yield of SL. As illustrated in Fig. 2, the optimal separation conditions of lignin were NaOH concentration of 40g L^−1^, liquid–solid ratio of 40 : 1 mL g^−1^, temperature of 60 °C and time of 2.5 h.

### 3.2 Optimization *via* RSM

The interaction effects of four independent variable on the yield of SL were studied. A total of 29 experiments were performed according to the best separation at optimal yield of lignin, and results of response surface were provided in Table S3.† Quadratic polynomial fitting was carried out for the test data in Table S3† with the design Expert software 8.0.6 version. The model fitting equation explained effects of four variables (*A*, *B*, *C* and *D*) on the response variable (*Y*) was shown below:

3 *Y* = −279.442 + 5.091*A* + 5.605*B* + 4.019*C* + 9.155*D* − 0.035*AB* + 0.028*AC* + 0.0005*AD* + 0.0348*BC* − 0.2315*BD* + 0.044*CD* − 0.0687*A*^2^ − 0.0757*B*^2^ − 0.0537*C*^2^ − 0.8566*D*^2^ (*R*^2^ = 96.53%)

where *A* = NaOH concentration, *B* = liquid–solid ratio, *C* = temperature and *D* = time.

The adequacy of the model was described by analysis of variance (ANOVA) and results were exhibited in Table 2. The obtained value (*P* < 0.05) for the model and lack of fit value (*P* > 0.05) indicated that the fitting of the model was statistically adequate and desirable to describe the response.

**Table 2:** Variance analysis results of response surface

	Sum of squares	Degree of freedom	Mean square	*F* value	Prob > *F*
Model	1040.23	14	74.30	27.84	<0.0001
*A*	10.45	1	10.45	3.92	0.0678
*B*	151.59	1	151.59	56.79	<0.0001
*C*	54.02	1	54.02	20.24	0.0005
*D*	7.19	1	7.19	2.69	0.1230
*AB*	49.84	1	49.84	18.67	0.0007
*AC*	32.60	1	32.60	12.21	0.0036
*AD*	0.00025	1	0.00025	0.0000936	0.9760
*BC*	48.44	1	48.44	18.15	0.0008
*BD*	5.36	1	5.36	2.01	0.1784
*CD*	0.19	1	0.19	0.073	0.7916
*A* ^2^	306.40	1	306.40	114.78	<0.0001
*B* ^2^	371.87	1	371.87	139.31	<0.0001
*C* ^2^	187.43	1	0.30	70.21	<0.0001
*D* ^2^	0.30	1	0.30	0.11	0.7434
Residual	37.37	14	2.67		
Lack of fit	35.02	10	3.50	5.96	0.0501
Pure error	2.35	4	0.59		
Total error	1077.60	28			

**Fig. 3:**
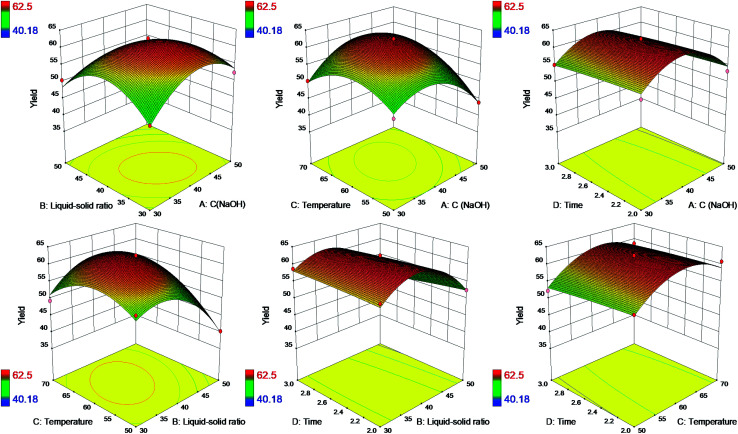
3D surface plot showing the interaction of four effects on yield of SL.

Response surface plots were exhibited in Fig. 3 based on the model eqn (3) to illustrate the interaction effects of four independent factors on the yield of SL.

The six plots demonstrated the relative effects of two factors by keeping the another two factors constant simultaneously. It could be found that the interaction effects of NaOH concentration and liquid–solid ratio, NaOH concentration and reaction temperature, liquid–solid ratio and reaction temperature had significant influence on the yield of SL. However, the time factor showed a narrow positive influence on the yield of SL. As a consequence, only three factors (NaOH concentration, liquid–solid ratio and temperature) are required to be selected as the optimal separation condition, and time could be as short as 2 hours. It's energy-saving and more efficient to separate alkaline lignin with higher yield at less time. Therefore, it has an advantage to utilize more alkaline lignin replacing petroleum-based polyether polyol to produce bio-based polyurethane foams on a large scale.

### 3.3 Quantitative ^31^P NMR analysis of lignin

**Fig. 4:**
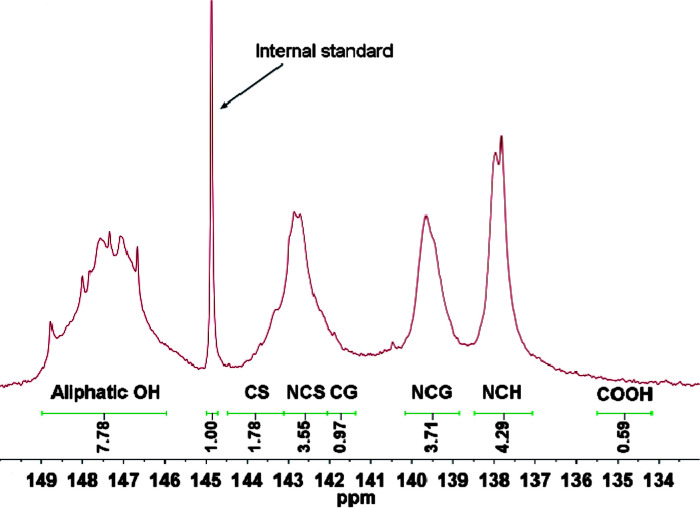
Quantitative ^31^P-NMR spectrum of separated lignin. CS, condensed syringyl OH; NCS, noncondensed syringyl OH; CG, condensed guaiacyl OH; NCG noncondensed guaiacyl OH; and NCH, noncondensed *p*-hydroxyphenyl OH.

^31^P NMR analysis has been widely applied in the characterization of hydroxyl groups in lignin. The phosphitylated hydroxyls could be quantitatively evaluated by an internal standard which describes adequate stability and satisfactory precision from lignin hydroxyl regions in a ^31^P NMR spectrum.^29^ A typical ^31^P NMR spectrum of separated lignin was illustrated in Fig. 4.

Typical chemical shifts/integration regions and hydroxyl group content in separated lignin using quantitative ^31^P NMR analysis was summarized in Table 3. It was easy to find that total hydroxyl group content of separated lignin was 22.08 mmol g^−1^, which was far higher than polyether polyol (1.06 mmol g^−1^). Moreover, separated lignin had bigger S/G (syringyl units/guaiacol units) ratio of 1.14 compared to dioxane-lignin (0.22).^19^ The ^31^P NMR data (Table 3) demonstrated that lignin isolated from enzymatic hydrolysis residues had the following order of hydroxyl contents: aliphatic OH > syringyl OH > guaiacyl OH > *p*-hydroxyphenyl OH > carboxylic OH, which was different with that of hardwood lignin.^29^

**Table 3:** Typical chemical shifts/integration regions and hydroxyl group content in separated lignin

	Aliphatic OH	Phenolic OH					
		CS	NCS	CG	NCG	NCH	COOH
*δ* (ppm)	146.0–149.0	143.2–144.5	142.1–143.2	141.4–142.1	138.8–140.2	137.1–138.5	134.2–135.5
OH (mmol g^−1^)	7.78	1.78	3.55	0.97	3.71	4.29	0.59

### 3.4 FT-IR analysis

**Fig. 5:**
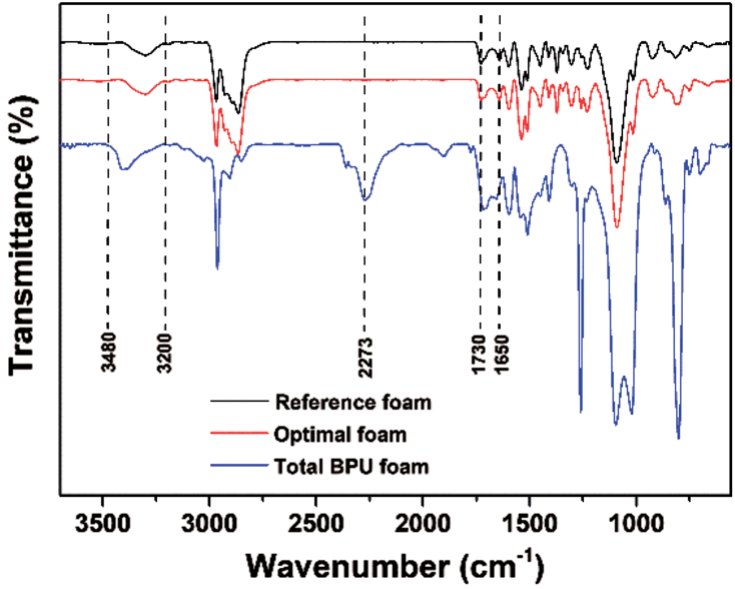
FT-IR spectra of BPU foams.

Considering the results of apparent density and mechanical properties of BPU foams, two foams with lignin content 0 and 15% were chosen as the reference foam and optimal foam, respectively, which prepared at the optimal conditions exhibited similar FT-IR spectra in Fig. 5. Specially, some total BPU foams were prepared for better comparison, whose conditions were the same as those of optimal foam excluding polyether polyol. The presence of urethane linkages of three PU foams could be demonstrated qualitatively by the FT-IR spectra because of the similar trend in spite of the different compositions. Obviously, the absorption bands at 3480–3200 cm^−1^ is associated with stretching vibration of –NH groups.^31^ The narrow band at 1730–1650 cm^−1^ is related to the characteristic vibration of –C=O groups, which are the essential carbon structure of polyurethane foams.^32^ These features indicated the occurrence of chemical interaction between the hydroxyl groups (separated lignin and polyether polyol) and MDI in three samples showed in Fig. 5.^31^ Interestingly, a peak at wavenumber 2273 cm^−1^ is assigned to unreacted –NCO group of the total BPU foam, which is attributed to the superfluous MDI.

### 3.5 Thermal stability of BPU foams

**Fig. 6:**
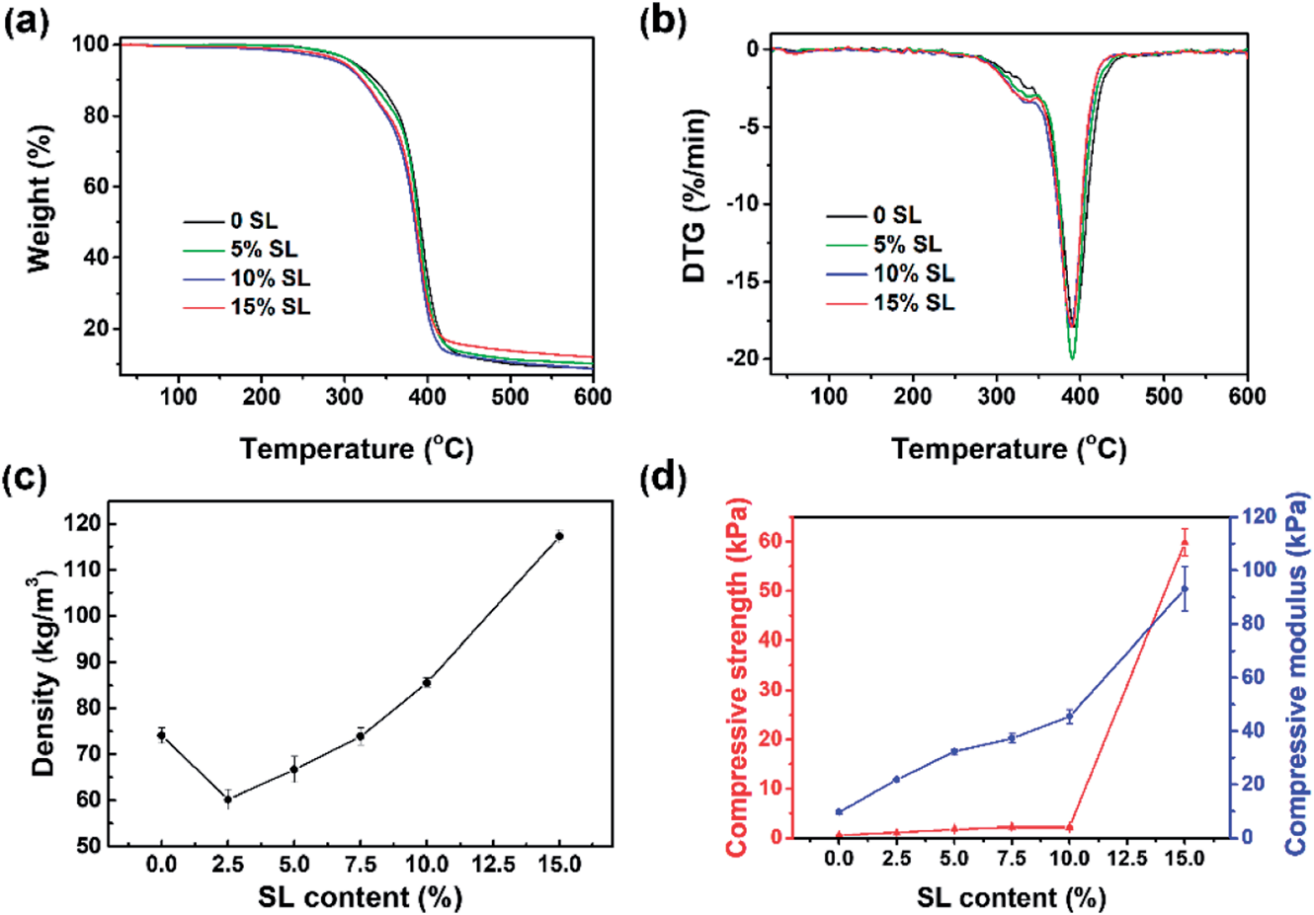
TG (a), DTG (b), density (c) and mechanical properties (d) of foams with different amounts of lignin.

Thermogravimetric analysis (TGA) was conducted to evaluate the influence of SL content on the thermal stability and compositional properties of the foams, which was closely related to the foam density and cell morphology.^15^ As demonstrated in Fig. 6a–b, the TG and DTG carves below 250 °C indicated that there was not much difference between BPU and the reference PU foams with respect to thermal stability. Three weight loss regions were observed in the TG and DTG curves. A small event observed in the TG curve prior to 300 °C (approximately 6% weight loss) is probably attributed to the loss of some unreacted small molecules.^7^ At the narrow range around 300–400 °C, all BPU foams had an intense degradation. The initial stage of weight loss at approximately 300 °C was attributed to the degradation of the polyol component and sample with 15% SL had a slightly higher decomposition rate than foams containing 5% SL and 0 SL, which indicated that the replacement of petroleum-based polyether polyol with alkaline lignin could facilitate the initial decomposition level. The second stage at around 400 °C was mainly assigned to the decomposition of urethane structures of the BPU foams.^31^ It is interesting to observe that the BPU foam with 15% SL had a narrowly larger weight loss and higher decomposition rate than the foams containing 5% SL and 0 SL, indicating that less urethane linkages formed in the BPU foams which involved more SL because the amount of accessible hydroxyl groups and active group in alkaline lignin is less than in petroleum-based polyether polyol.^31^ For BPU foams after 400 °C, weight loss were considerably small and even could be negligible. Consequently, the results suggested that the thermal stability of the BPU foams was slightly reduced by the addition of the SL.

### 3.6 Apparent density

Effect of SL content on the apparent density of BPU foams was illustrated in Fig. 6c, and these density values were in agreement with the literature data for foams containing lignin.^7,24^ The result indicated that a remarkable enhancement of apparent density observed upon increasing the SL content from 2.5% to 15%. Densities of the foams with 2.5% SL are similar *i.e.*, 60.14 (±2.16) kg m^−3^. However, the BPU foams prepared by directly replacing 15% of petroleum-based polyether polyol with SL have higher densities 117.30 (±1.43) kg m^−3^. A potential reason for this highly enhanced foam density is a relatively lower hydroxyl reactivity of SL, which contributes to a slower polyaddition reaction rate. A slower polyaddition reaction rate leads to more CO_2_ gases escaping from the foam structure and hence smaller void volumes and higher foam densities.^24^ Similar results have been reported in literatures,^4,32^ which suggested that the polymeric chains were more packaged with smaller cells and less CO_2_ gas produced by the polyaddition reaction.^7^ Interestingly, the density of BPU foams prepared with 2.5–7.5% SL are lower than that of the reference foams. This result was probably because the few addition of lignin increased the percent of heterogeneous cells and the reaction time that could result in much more loss of CO_2_ during the polymerization.

### 3.7 Mechanical properties

Compressive strength and modulus are the representative parameters of the firmness, which is one of the most important characteristics of the foams together with their density. Fig. 6d showed that the compressive modulus increased by an astonishing 9.56 times from 9.74 kPa to 93.14 kPa with SL content increasing from 0 to 15%. Moreover, the compressive strength boosted exponentially up to 59.87 kPa when SL content came to 15%, which could be attributed to higher content of lignin considered as adhesive compound and filling agent. However, the compressive strength enhanced narrowly from 0.61 kPa to 2.17 kPa as the lignin content increased from 0 to 10%, which could be attribute to the relative low amount of hydroxyl groups in lignin leading to the low cross-linking density and small compressive strength of the BPU foams as compared to pure polyol. Furthermore, alkaline lignin was not absolutely mixed with the petroleum-based polyether polyol. Consequently, the incorporation of a heterogeneous mixture of lignin and polyol also led to an irregular cellular structure as demonstrated by following SEM images. These results could be explained by the fact that alkaline lignin macromolecules were inclined to agglomerate, self-associate, and form the interpenetrating polymer network rather than chemical interaction with polyurethane chains when excessive amounts of alkaline lignin were added into the polyurethane foam matrix.^31^ Compressive strength and modulus of BPU foams containing 15% alkaline lignin which was directly physically mixed with polyether polyol without liquidation, heating, microwave treatment and chemical modification are far more excellent than other foams^7,32^ which contained the same content of lignin. This increase trend was in agreement with some previous work.^32,33^

### 3.8 Morphology of BPU foams

**Fig. 7:**
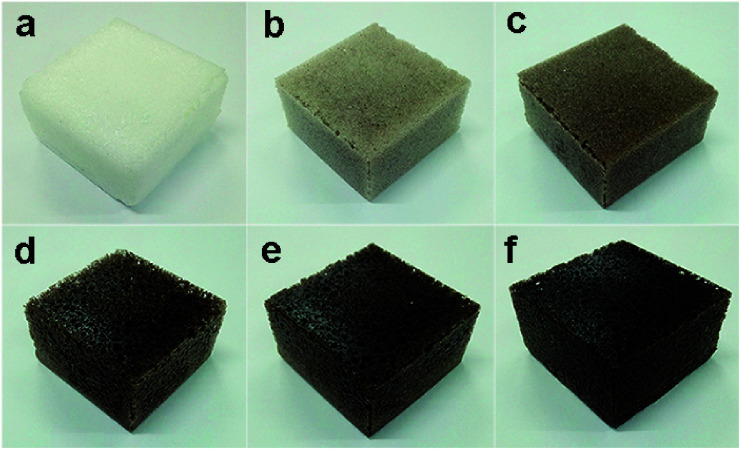
Macro photographs of BPU foams (50 × 50 × 25 mm) with different content of SL.

**Fig. 8:**
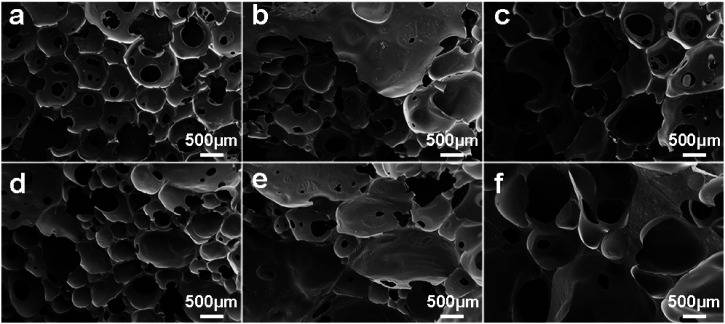
Macro photographs of BPU foams with different content of SL: (a) 0, (b) 2.5%, (c) 5.0%, (d) 7.5%, (e) 10%, and (f) 15%.

The Macro photographs for BPU foams with different content of SL were illustrated in Fig. 7. It can be seen that foams in Fig. 7d–f with high lignin content had dark color and slightly crude surface compared with white color and smooth surface of the reference foam (Fig. 7a). With increasing the lignin content, the cellular shape became more heterogeneous and irregular along with extra formation of large cells, and the color of foams became darker. As exhibited in Fig. 8a–f, the cells of BPU foams showed a honeycomb structure with uniform and smooth surface texture. Additionally, cell wall of BPU foam with 15% SL were much thicker and less homogeneous than that of the reference foam, because the heterogeneous mixture of lignin and polyol is less expandable and results in a faster foaming reaction, and hence the evolution of CO_2_ gas *via* cracked cells.

This alteration in cell morphology was probably attributed to the fact that the SL may affect the process of cell nucleation in preparation of BPU foam.^24^ Two significant parameters that make a great difference on the mechanical properties of foams are thickness and length of cell shape, which is related to amount of lignin due to its aromatic rings which led to an increase in chain stiffness.^33^ Specifically, thicker cell membrane could contribute to greater compressive strength and modulus, corresponding with the results of mechanical properties.

In general, the BPU foams prepared by partially replacing petroleum-based polyether polyol with SL exhibited less homogeneous and regular cell structure than the reference foams utilizing the pure petroleum-based polyether polyol. Consequently, the preparation of BPU foams employing lignin from corn stalk enzymatic hydrolysis residues needs further optimization of surfactant, catalysts and blowing agents to improve the homogeneity of BPU foams.

## 4 Conclusion

This paper reports a low-cost, green and relatively simple synthesis of bio-based polyurethane foams employing separated lignin without any further surface chemical modification. Lignin was extracted from enzymatic hydrolysis residues with an easy alkaline treatment and the optimal conditions were NaOH concentration of 39.90 g L^−1^, liquid–solid ratio of 38.75 : 1 mL g^−1^, reaction temperature of 61.33 °C, and reaction time of 2 h. Furthermore, RSM results showed the interaction effect of NaOH concentration and liquid–solid ratio, NaOH concentration and reaction temperature, liquid–solid ratio and reaction temperature had significant influence on the yield of lignin. 9.56 times increase in the compressive modulus and 97.93 times boost in the compressive strength of BPU foams were due to the content of 15% SL. The prepared foams with higher lignin content exhibited an obviously increase in the apparent density and mechanical properties but a little decrease in thermal stability. The use of this bio-based polyol from industrial residual sources replacing partial oil-based polyol not only provides a high sustainability for polyurethane foam material but also helps to make the most of waste from the cellulosic ethanol industry which can contribute to the economic feasibility. In addition, these environmentally friendly bio-based polyurethane foams exhibited good mechanical properties and potential for utilization on the industrial scale and in various fields of application.

## Conflicts of interest

There are no conflicts to declare.

## Supplementary Material

RA-008-C8RA01089G-s001
