# Supplementary material for: Bio-based polyurethane foam preparation employing lignin from corn stalk enzymatic hydrolysis residues
Source: RSC Adv. 2018 Apr 26;8(28):15754–61. doi: 10.1039/c8ra01089g (PMC9080235; doi:10.1039/c8ra01089g)
Supplement: RA-008-C8RA01089G-s001 [file RA-008-C8RA01089G-s001.pdf]

Electronic Supplementary Material (ESI) for RSC Advances.
This journal is © The Royal Society of Chemistry 2018

Electronic Supplementary Information

Bio-based Polyurethane Foam Preparation Employing Lignin from Corn Stalk Enzymatic Hydrolysis Residues

Shiyun Zhu,[ab] *Kefu Chen*,[ab] *Jun Xu*,*[ab] *Jun Li*,[a] *and Lihuan Mo*[a]

[a]State Key Laboratory of Pulp and Paper Engineering, South China University of Technology，Guangzhou 510640，Guangdong，China

[b]Plant Micro/Nano Fiber Research Center, South China University of Technology，Guangzhou 510640，Guangdong，China

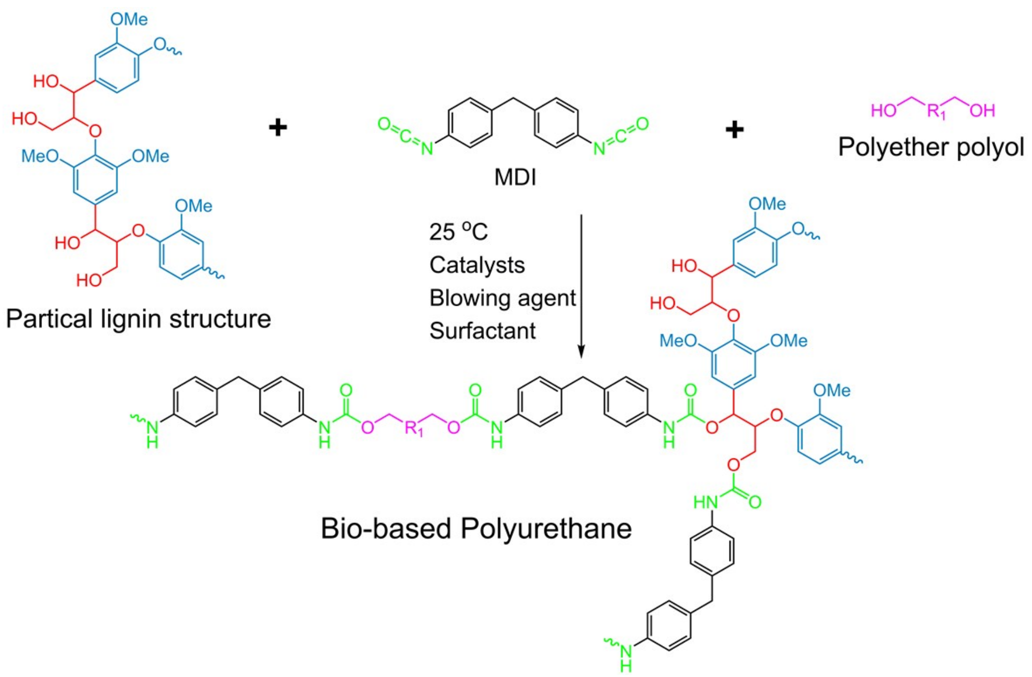

Fig. S1 Reaction of the synthesis of BPU foams.

Table S1 Experimental conditions of single factor

factor	Level				
	1	2	3	4	5
C(NaOH) (g/L)	20	30	40	50	60
L-S ratio (mL/g)	20:1	30:1	40:1	50:1	60:1
Temperature(^{o}C)	30	40	50	60	70
Time (h)	1.5	2	2.5	3	3.5

Table S2 Box-Behnken design for experiments

Factor	Number	Level		
		-1	0	1
C(NaOH) (g/L)	A	30	40	50
L-S ratio (mL/g)	B	30:1	40:1	50:1
Temperature(^{o}C)	C	50	60	70
Time (h)	D	2	2.5	3

Table S3 Experimental design and results of response surface

Number	factor				Y/%
	NaOH concentration	liquid-solid ratio	temperature	time	
1	-1	0	0	1	55.02
2	0	-1	0	-1	58.27
3	0	-1	0	0	62.50
4	-1	0	1	0	50.35
5	-1	0	-1	0	50.12
6	1	1	1	0	52.41
7	0	0	-1	-1	55.44
8	0	-1	0	1	58.68
9	0	0	0	0	62.5
10	1	0	0	1	53.21
11	0	1	-1	0	40.25
12	0	0	1	-1	60.79
13	0	-1	-1	0	55.31
14	0	0	-1	1	52.31
15	1	0	0	0	53.21
16	-1	1	0	-1	50.48
17	0	1	0	1	48.31
18	-1	-1	0	0	48.59
19	0	0	0	0	62.50
20	-1	0	0	-1	55.12
21	1	0	1	0	55.56
22	0	0	1	1	58.54
23	0	0	0	0	61.1
24	1	1	0	0	40.18
25	0	-1	1	0	49.35
26	1	0	-1	0	43.91
27	0	1	1	0	48.21
28	0	0	0	0	61.10
29	0	1	0	-1	52.53
